# Plasma intact fibroblast growth factor 23 levels in women with bulimia nervosa: A cross-sectional pilot study

**DOI:** 10.1186/1751-0759-5-7

**Published:** 2011-06-17

**Authors:** Makoto Otani, Yoshiyuki Takimoto, Junko Moriya, Kazuhiro Yoshiuchi, Akira Akabayashi

**Affiliations:** 1 Department of Stress Sciences and Psychosomatic Medicine, The University of Tokyo, 7-3-1 Hongo, Bunkyo-ku, Tokyo 113-8655, Japan; 2 Department of Psychiatry, NTT Medical Center Tokyo, 5-9-22 Higashi-Gotanda, Shinagawa-ku, Tokyo 141-8625, Japan

**Keywords:** fibroblast growth factor 23 (FGF23), intact fibroblast growth factor 23 (iFGF23), eating disorders, bulimia nervosa (BN), binge eating, frequency of binge eating, dietary phosphate, plasma phosphate, 1,25-dihydroxyvitamin D (1,25-(OH)_2_D)

## Abstract

Fibroblast growth factor (FGF) 23, a circulating 26-kDa peptide produced by osteogenic cells, is a novel phosphaturic factor. In our previous study, binge-eating/purging type anorexia nervosa (AN-BP) patients had elevated plasma intact FGF23 (iFGF23) levels, while restricting type (AN-R) patients had plasma iFGF23 levels similar to healthy controls. Although bulimia nervosa (BN) patients as well as some patients with AN-BP regularly engage in binge eating, there have been no studies regarding plasma iFGF23 levels in BN patients. Therefore, this study was performed to determine plasma iFGF23 concentrations in BN patients and healthy controls. The study population consisted of 13 female BN patients and 11 healthy female controls. Blood samples were collected from all subjects after overnight fasting. Plasma iFGF23 was measured using an ELISA kit in a cross-sectional manner. The two-tailed Mann-Whitney U-test was used to assess differences between BN patients and healthy controls. In addition, BN patients were divided into two groups based on questionnaire-reported binge eating frequency immediately prior to participation in this study: high frequency of binge eating (once a week or more; HF group; *n *= 8) and low frequency of binge eating (less than once a week; LF group; *n *= 5). Two-tailed Mann-Whitney U-test with Bonferroni's correction was performed after the Kruskal-Wallis test to assess differences between HF group, LF group, and healthy controls. Median (quartiles) plasma iFGF23 levels were greater in BN patients (35.5 [14.8-65.0] pg/ml) than in controls (3.8 [not detected-5.3] pg/ml; p = 0.002). In addition, median (quartiles) plasma iFGF23 levels were greater in the HF group (62.3 [44.4-73.4] pg/ml) than in controls (p < 0.001) and in the LF group (12.9 [not detected-30.3] pg/ml; p = 0.011), while there were no differences between the LF group and controls (p = 0.441). This is the first study to show that BN patients have elevated plasma iFGF23 levels. Moreover, this study showed that BN patients with a high frequency of binge eating have elevated plasma iFGF23 levels, while iFGF23 levels are similar to healthy controls in those with a low frequency of binge eating. Plasma iFGF23 level may be a suitable indicator of binge eating in BN patients.

## Findings

Fibroblast growth factor (FGF) 23, a circulating 26-kDa peptide produced by osteogenic cells, is a novel phosphaturic factor, which is important for the regulation of inorganic phosphate homeostasis and for vitamin D metabolism [1]. FGF23 inhibits renal proximal tubule phosphate reabsorption, increases renal phosphate excretion, and reduces serum phosphate without affecting serum calcium. FGF23 also strongly suppresses 1,25-dihydroxyvitamin D (1,25-(OH)_2_D) production [2,3].

Circulating FGF23 levels are regulated by serum phosphate [4,5], 1,25-(OH)_2_D [3,6] and dietary phosphate [6]. Dietary phosphate plays an important role in FGF23 regulation, and dietary phosphate loading increases circulating FGF23 levels in healthy men in a matter of days [7], even without changes in serum phosphate or 1,25-(OH)_2_D levels [8].

Bulimia nervosa (BN) is an eating disorder characterized by habitual binge eating, inappropriate compensatory behaviors, such as self-induced vomiting, a preoccupation with body weight, and excessive self-evaluation of weight and shape. For BN, the Diagnostic and Statistical Manual of Mental Disorders-Fourth Edition (DSM-IV) specifies that binging and compensatory behaviors must occur with a minimum average frequency and duration of at least twice a week for three months [9].

Our previous study showed that binge-eating/purging type anorexia nervosa (AN-BP) patients had elevated plasma intact FGF23 (iFGF23) levels, while restricting type (AN-R) patients had plasma iFGF23 levels similar to healthy controls [10]. Although BN patients as well as some patients with AN-BP regularly engage in binge eating, there have been no previous studies regarding plasma iFGF23 levels in BN patients. Therefore, the present study was performed to determine plasma iFGF23 concentrations in BN patients and healthy controls.

The study population consisted of 13 female outpatients of The University of Tokyo Hospital diagnosed with purging type BN according to DSM-IV by experienced clinicians, and 11 healthy female controls. All of the patients had binge eating episodes at least twice a week on average within the last three months, while the frequencies of binge eating episodes in some of the patients were small within the last two to four weeks just before the study because they were undergoing treatment. In addition, BN patients were divided into two groups on the basis of questionnaire-reported binge eating frequency just prior to participation in this study: patients with high frequency of binge eating (once a week or more; HF group; *n *= 8) and patients with low frequency of binge eating (less than once a week; LF group; *n *= 5). Physical comorbidity and medication directly affecting calcium and phosphate metabolism at the time of enrollment in this study were exclusionary criteria.

Blood samples were collected from all participants after overnight fasting. All BN patients completed a questionnaire about frequency of binge eating and vomiting for two weeks to one month just prior to participation in this study. Frequency of binge eating was rated on a six-point scale as follows: "hardly ever," "once a month," "once a week," "two or three times a week," "daily," and "two or three times a day." Frequency of vomiting was rated on a seven-point scale as follows: "never," "occasionally," "once a week," "two or three times a week," "daily," "two or three times a day," and "more than four times a day." The protocol was approved by the Institutional Ethics Committee of the University of Tokyo, and written informed consent was obtained from all subjects prior to enrollment in the study.

All blood samples were drawn into chilled tubes containing EDTA-2Na (1 mg/ml) and were then immediately centrifuged at 4°C. Plasma portions were stored at -70°C prior to analysis. Plasma concentrations of iFGF23 were measured using an ELISA kit (Immutopics, San Clemente, CA) [10-12], with a sensitivity of 1.0 pg/ml, intraassay variability of <4.4%, and interassay variability of <6.5%. All samples were analyzed in duplicate. Plasma 1,25-(OH)_2_D and 25-hydroxyvitamin D (25-OHD) concentrations were measured by RIA (SRL, Tokyo, Japan). Plasma calcium, phosphate, and intact parathyroid hormone (iPTH) concentrations were measured using standard laboratory methods (SRL).

The two-tailed Mann-Whitney U-test was used to assess the significance of differences between BN patients and healthy controls. The two-tailed Mann-Whitney U-test with Bonferroni's correction was performed after the Kruskal-Wallis test to assess the significance of differences between the HF group, LF group, and healthy controls. Values of p < 0.05 were considered significant, except that p < 0.017 was considered significant in the two-tailed Mann-Whitney U-test with Bonferroni's correction. Spearman's rank-correlation coefficients (ρ) were used to assess the relationship between plasma iFGF23 levels and plasma phosphate or 1,25-(OH)_2_D levels for BN patients. All statistical calculations were performed using SPSS for Windows version 10.0 (SPSS, Chicago, IL). All data are presented as the median, first quartile, and third quartile.

**Figure 1:**
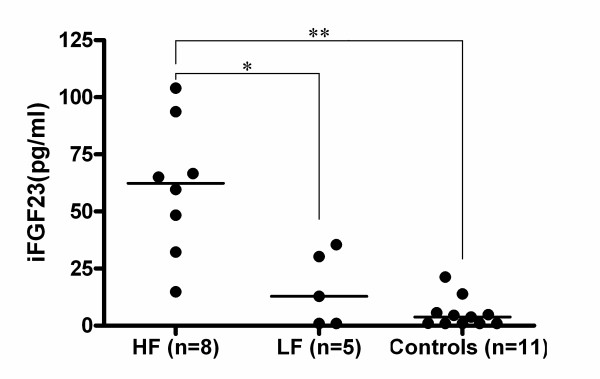
**Dot plots of plasma intact fibroblast growth factor 23 (iFGF23) levels in bulimia nervosa (BN) patients with a high frequency of binge eating (HF group), BN patients with a low frequency of binge eating (LF group), and healthy controls**. The graphs show median values (bars). The two-tailed Mann-Whitney U-test with Bonferroni's correction was used to assess the significance of differences among groups. A value of p < 0.017 was considered statistically significant. *p < 0.017 vs. LF group. **p < 0.001 vs. controls.

Clinical profiles and biochemical data are summarized in Table 1. Median (quartiles) plasma iFGF23 levels were significantly greater in all BN patients (35.5 [14.8-65.0] pg/ml) than in controls (3.8 [not detected-5.3] pg/ml; p = 0.002). In addition, median (quartiles) plasma iFGF23 levels were significantly greater in the HF group (62.3 [44.4-73.4] pg/ml) than in healthy controls (p < 0.001; Figure 1) or the LF group (12.9 [not detected-30.3] pg/ml; p = 0.011; Figure 1), while there were no significant differences between the LF group and healthy controls (p = 0.441; Figure 1). Binge eating behavior was significantly more frequent in the HF group than in the LF group (p = 0.002), while there were no differences in frequency of vomiting between the HF group and the LF group (p = 0.222).

**Table 1:** Clinical profiles and biochemical data of women with bulimia nervosa and healthy controls

	BN patients with high frequency of binge eating (n = 8)	BN patients with low frequency of binge eating (n = 5)	Controls (n = 11)	Normal values	p
Body Mass Index (kg/m^2^)	19.9 (18.4-22.0)	18.6 (18.2-18.6)	19.7 (19.5-21.8)		0.103
Age (years)	28 (25-28)	25 (23-27)	27 (21-29)		0.792
age at the time of disease onset (years)	19 (18-21)	19 (18-20)			
disease duration (years)	5.8 (4.0-9.8)	6.0 (3.5-9.0)			
frequency of binge eating	2-3/week (2-3/week-1/day)*	1/month (1/month-1/month)			
frequency of vomiting	2-3/week (occasionally-1/day)	occasionally (occasionally-occasionally)			
uncorrected Ca (mg/dl)	8.8 (8.7-9.3)	9.4 (9.2-9.7)	8.9 (8.7-9.1)	8.5-10.2	0.204
P (mg/dl)	3.6 (2.9-4.1)	3.5 (2.9-3.6)	2.9 (2.5-3.2)	2.4-4.3	0.133
intact PTH (pg/ml)	62 (43-69)	54 (45-55)	38 (33-45)	10-65	0.073
1,25-(OH)_2_D (pg/ml)	39.4 (17.1-55.6)	38.2 (37.7-42.9)	39.7 (20.9-47.5)	20.0-60.0	0.837
25-OHD (ng/ml)	15 (13-16)	18 (17-19)	20 (15-21)	7-41	0.186

All values are shown as the median (first quartile-third quartile). The p-values in the rightmost column were calculated using the Kruskal-Wallis test.

* p < 0.05 vs. BN patients with low frequency of binge eating (two-tailed Mann-Whitney U-test)

BN; bulimia nervosa. PTH; parathyroid hormone. 1,25-(OH)_2_D; 1,25-dihydroxyvitamin D. 25-OHD; 25-hydroxyvitamin D.

For all BN patients, iFGF23 levels were correlated with neither plasma phosphate levels (ρ = 0.160, p = 0.602) nor plasma 1,25-(OH)_2_D levels (ρ = 0.132, p = 0.667).

This is the first study to show that BN patients have elevated plasma iFGF23 levels. Moreover, the present study showed that BN patients with a high frequency of binge eating have elevated plasma iFGF23 levels, while the iFGF23 levels are similar to healthy controls in those with a low frequency of binge eating. These results suggest that plasma iFGF23 levels may be associated with binge eating frequency in BN patients. Gwirtsman et al. [13] showed that frequent vomiting increased serum amylase levels in BN patients. The serum amylase level is an established indicator of vomiting behavior. However, there are no established indicators of binge eating behavior. The establishment of an indicator of binge eating in BN patients would be therapeutically useful. Plasma iFGF23 level may be a suitable indicator of binge eating behavior in BN patients.

During binge eating, BN patients eat a large quantity of food at once, including foods such as chocolates, cakes, snacks, and sweet buns, which generally contain moderate to large amounts of phosphate. Thus, binge eating in BN patients may be regarded as dietary phosphate loading. It is speculated that dietary phosphate loading with binge eating may increase plasma iFGF23 levels in BN patients. The present study showed that there were no significant differences in plasma calcium, phosphate, iPTH, 1,25-(OH)_2_D, or 25-OHD levels between the three groups. These results were consistent with the speculation.

The present study had three limitations. First, the number of BN patients was small. Second, the volumes of binge eating and purging in the BN patients prior to participation in the study were not available. Third, dietary phosphate intake prior to participation in the study was not assessed in BN patients. We were therefore unable to completely assess the relationships between binge eating behavior, dietary phosphate intake, and plasma iFGF23 levels. In future studies, dietary phosphate intake and the volume of binge eating prior to participation in the study should be determined in addition to plasma iFGF23, 1,25-(OH)_2_D, and 25-OHD levels.

This preliminary study showed that BN patients have elevated plasma iFGF23 levels, and that BN patients with a high frequency of binge eating have elevated plasma iFGF23 levels, while iFGF23 levels are similar to healthy controls in those with a low frequency of binge eating. Plasma iFGF23 level may be a suitable indicator of binge eating in BN patients.

## Competing interests

The authors declare that they have no competing interest.

## Authors' contributions

MO designed the study, analyzed the data, performed the statistical analysis, interpreted the results, and drafted the manuscript. JM collected the data. YT, KY, and AA helped analyze the data, interpret the results, and draft the manuscript. All authors have read and approved the final manuscript.
